# [Retracted] MicroRNA-150 upregulation reduces osteosarcoma cell invasion and metastasis by downregulating Ezrin

**DOI:** 10.3892/ol.2026.15471

**Published:** 2026-01-26

**Authors:** Ce Zhan, Cheng Li, Hao Zhang, Hao Tang, Fang Ji, Su-Chi Qiao, Wei-Dong Xu, Zhi-Wei Wang

Oncol Lett 12: 3457–3462, 2016; DOI: 10.3892/ol.2016.5046

Following the publication of this paper, it was drawn to the Editor's attention by a concerned reader that, regarding the Transwell assay data shown in Fig. 2, panels A, B, and E all appeared to be showing the same sample, albeit rotated at various angles in these panels, suggesting that data which were intended to show the results of differently performed experiments had been derived from the same original source. In addition, the same data sample appeared subsequently in a paper written by different authors that was published in the journal *Bioscience Reports*, which has since been retracted. Furthermore, an analysis of the data in this paper performed by the Editorial Office revealed that these data in Fig. 2, and the western blot data in Fig. 1, had subsequently appeared in a number of other articles that were written by different authors at different research institutes, several of which have likewise been retracted.

Owing to the number of overlapping data panels identified in Fig. 2, and given the fact that the contentious data in the above article have been found to have been featured in a number of subsequently published articles, the Editor of *Oncology Letters* has decided that this paper should be retracted from the Journal on account of a lack of confidence in the presented data. The authors were asked for an explanation to account for these concerns, but the Editorial Office did not receive a reply. The Editor apologizes to the readership for any inconvenience caused.

